# E-fast in patients with dengue infection in medical ward: a pilot study

**DOI:** 10.1186/2036-7902-7-S1-A26

**Published:** 2015-03-09

**Authors:** A Abdul Rahim, M Abd-Rahman, SZ Zulkifli, SS Md Sani

**Affiliations:** 1 Fellow in Acute Internal Medicine, Department of Medicine, Hospital Kuala Lumpur, Malaysia; 2 Acute Internal Medicine Specialist, Department of Medicine, Hospital Kuala Lumpur, Malaysia

**Keywords:** Pleural Effusion, Fluid Collection, Warning Sign, Severe Illness, Dengue Fever

## Background

Dengue is a major tropical infection in Malaysia. It has several presentations and may progress to severe illness. Mortality is high if left unsupported. Warning Signs (WS) ; nausea, vomiting abdominal pain, bleeding and leaking syndrome (LS) (ascites and pleural effusion) are crucial in dengue assessment. Abdominal pain and tenderness, gastrointestinal bleed, jaundice, hepatomegaly and ascites are predictors of severe illness and require intensive care support as mentioned in the literatures.

At present, E-FAST is used widely in emergency unit to aid diagnosis and management in acute emergency and trauma. It has not been used in dengue infection to detect leaking syndrome. The clinical sign of leaking syndrome can be subtle if minimal. Early detection will lead to closer monitoring and anticipation of further change of clinical condition, hence aid in management of dengue.

## Objective

To assess the utility of E-FAST in detecting LS in dengue infection.

## Patients and methods

Cross-sectional study of patients with dengue infection admitted to medical wards in The Department of Medicine Hospital Kuala Lumpur from 18 August 2014 to 30 August 2014. All dengue infection cases received standard treatment. E-FAST was performed by a group of 4 physicians trained by WINFOCUS Malaysia Team. Each scan was performed by a physician, and reviewed by another . All baseline information, clinical and scan findings were recorded.

## Results

Total of 21 patients were observed; 13 (62%) males and 8 (8%) females, with median age of 24 years old ( *IQR 73*). Median days for presentation of illness were 4 days ( *IQR 8*). 4 (19%) patients presented in defervescence phase and 17 (81%) patients presented in febrile phase. WS were observed in all patients in this study; 3 (14%) patients had 1 WS, 7 (33%) patients had less than 3 WS( < 3 WS) and 11 (52%) had 3 and more WS (≥ 3WS ) . On discharge, 14 (67%) patients had diagnosis of dengue fever with WS and 7 ( 33%) patients had diagnosis of severe dengue.

LS were detected in total of 9 out of 21(43%) patients. 3 out of 9 (33%) patients were clinically detected by clinician versus 6 out of 9 (67%) patients by E-FAST (p < 0.05). 5 patients had pleural effusion; 3 patients detected by clinician and 5 patients detected by E-FAST ( p < 0.05). 9 patients had ascites in which 1 detected by clinician and 9 detected by E-FAST ( p = 0.237). In those with LS , 8 ( 88%) patients had ≥ 3 WS compared to 1 (11%) patients had < 3 WS ( p < 0.05). In LS 7 (78%) patients had diagnosis of severe dengue on discharge and 2 (22%) patients had diagnosis of dengue fever with WS on discharge ( p < 0.05). In those with LS, 3 (33%) patients needed ICU admission and 6 (67%) was managed in general ward (p = 0.149).

## Conclusion

E-FAST is useful to detect fluid collection for LS in dengue patients with warning signs in Medical ward. Detection of LS is significantly better with E-FAST compare to clinical detection. Larger cohort of study population is needed for further evaluation of E-FAST usage in diagnosis, monitoring and management of dengue infection.

## References

[1] Ahmad Nizal MG RH, Mazrura S (2012). Dengue infections and circulating serotypes in Negeri Sembilan, Malaysia. Malaysian Journal of Public Health Medicine.

[2] Jamaiah I, RM, Nissapatorn V (2005). Prevalence of dengue fever and dengue hemorrhagic fever in Hospital Tengku Ampuan Rahimah, Klang, Selangor, Malaysia. Southeast Asian J Trop Med Public Health.

[3] Ooi ET, GS, Anil R (2008). Gastrointestinal manifestations of dengue infection in adults. Med J Malaysia.

[4] Fadilah S, SAW, Sahrir S, Mazlam MZ (2000). A comparison of the pattern of liver involvement in dengue hemorrhagic fever with classic dengue fever. Southeast Asian J Trop Med Public Health.

[5] Norlijah O, KN, Kamarul AR: **Clinico-laboratory profile of dengue haemorrhagic fever in Malaysian children.** *Asian-Oceanian Journal of Pediatrics and Child Health* 2004., **3**(2):

[6] Rozycki GS, Pennington Scott D., Feliciano David V (2001). Surgeon-Performed Ultrasound in the Critical Care Setting: Its Use as an Extension of the Physical Examination to Detect Pleural Effusion. Journal of Trauma-Injury Infection & Critical Care.

[7] Bouhemad Bélaïd, MZ, Lu Qin, Rouby Jean-Jacques: **Clinical review: Bedside lung ultrasound in critical care practice.** *Critical Care* 2007., **11**(205):10.1186/cc5668 PMC2151891 17316468

